# Interfacial Tension as a Parameter to Assess Demulsifier Efficiency on Heavy Crude Oil Emulsions

**DOI:** 10.1021/acsomega.4c06425

**Published:** 2025-08-01

**Authors:** Isabella. G. Freitas, Carlos. E. Perles, Vanessa. C. B. Guersoni, Tatiana M. Pessanha, Antonio. C. Bannwart

**Affiliations:** † Universidade de Campinas (UNICAMP), Dept. of Mechanical Engineering, Rua Mendeleyev, 200 − Cidade Universitária, 13083-860 Campinas, Brazil; ‡ Universidade de Campinas (UNICAMP), Center for Energy and Petroleum Studies, Cora Coralina, 350 − Cidade Universitária, 13083-896 Campinas, Brazil

## Abstract

The formation of water-in-crude oil (w/o) emulsions during the lifting and pipelining of crude oils is a common issue in petroleum production. In oilfields, emulsions are undesirable due to the increase of fluid viscosity, which consequently drops the production rate. Demulsifiers may be injected at electrical submersible pumps, production lines, and/or the crude oil processing station to deal with the impacts. In a laboratory, emulsion stability and demulsifier efficiency are commonly evaluated by bottle tests, but this approach does not consider the main factors controlling the kinetic stability of water–oil emulsions such as interfacial tension. This study aims to evaluate how the interfacial tension can influence the stability of these w/o emulsions, seeking a microscopic understanding of their phase separation as a function of temperature and demulsifier concentration. The Central Composite Rotatable Design (CCRD) methodology was used to evaluate the effects of the independent variables (temperature and demulsifier concentration) on the interfacial tension and emulsion stability at 50% water-cut. The interfacial tension was determined by a Spinning Drop Tensiometer, and the emulsion stability was determined by measuring the phase separation under a centrifugal field rather than the classical bottle test. We found an intrinsic inverse correlation between the yield of phase separation and interfacial tension, suggesting interfacial tension is an important parameter to assess the demulsifier’s effectiveness and the emulsion’s stability. Our studies surface important new findings for understanding the stability of emulsions for complex and more realistic crude oil–water systems with commercial demulsifiers.

## Graphical abstract

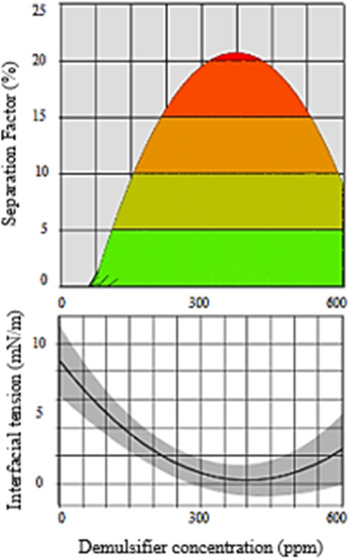

## 1 Introduction

Emulsions are systems in which two immiscible liquids, such as oil and water, are mixed to produce a fine dispersion of one into another. During the production and transportation of petroleum, the high temperature contributes to the emulsion formation, especially in water-in-crude oil type (w/o), because of the vigorous agitation, and viscosity reduction of the phases when subjected to high shear and turbulence conditions along the lifting/transportation lines.^1,2^

The emulsions are stabilized by natural surfactants in crude oil, such as asphaltenes, resins, and other amphiphilic species. The stabilization may be related to the smaller water droplets formed when natural surfactants have better adsorption at the water–oil interface. In addition, the apparent viscosity of the oil increases with the natural surfactant concentration increasing at the interfacial area.^3^ However, this scenario is undesirable because it negatively affects the performance of electric submersible pumps (ESP) and fluid flow through production lines.^1,2^ Given these problems in oil production, the injection of a demulsifier into the flooding fluid is considered one of the most effective means to improve oil recovery because it reduces the water–oil interfacial tension, enhancing the crude oil migration in rock pores, beyond speeding up the coalescence and phase separation in the separator, improving the oil quality for the following refining steps.^4–7^ Focusing on kinetic stability, the spontaneous formation of thin films of surfactants on the droplet interface is largely at fault for resistance against the thinning process.^8^

The relative emulsion stability may be predicted using the hydrophilic–lipophilic deviation (HLD) parameter, which is associated with the surfactant partition coefficient in three-phase systems (water–oil-surfactant). According to eqs 1 and 2, the HLD of a surfactant–water–oil system can be represented as a linear sum of the system’s formulation variations, considering the nature of the surfactant, oil characteristics, temperature, and salinity.^1,3^

1 $$HLD=\ln S-kACN-f\left(A\right)+\sigma -\alpha_{t}\Delta T$$

2 $$HLD=\mathrm{bS}-kACN-f\left(A\right)+\beta +c_{t}\Delta T$$

In the equations, *S* is the salinity of the aqueous phase in % NaCl, ACN is the number of carbons in the alkane (characteristic parameter of the oil phase), Δ*T* is the reference temperature (generally 25 °C) and *f*(*A*) is a linear function that considers the effect of the type and concentration of alcohol (cosurfactant). The coefficients *σ, β, b, k*, α*t*, and *ct* represent constants associated with the type of surfactant. For ionic surfactants, the HLD of the system is calculated from eq 1, and for nonionic surfactants, the HLD is calculated from eq 2.^1,3^

The HLD is a thermodynamic parameter that represents the equilibrium state of the whole system. The emulsion stability changes proportionally to the IFT and, consequently, with the HLD.^3,4^ Even though the stability is kinetic, the condition of the interface at equilibrium, indicated by IFT equilibrium values, can be considered a good parameter to evaluate how the affinity of the surfactant for the interface directly impacts the coalescence directly.

Emulsions reach the lowest stability at HLD = 0, a condition under which the surfactant has the same chemical potential in both phases at ambient temperature and pressure. At this HLD point, the interfacial tension also reaches the minimum value.^1,9^ The poor emulsion stability at HLD = 0 is explained since the fast exchange of demulsifier molecules between bulk and interface, minimizing the Gibbs–Marangoni effect.^10–12^

Great advances in the formulation of emulsions for oil recovery have been made since the 1970s and interfacial rheological properties of these systems have been studied for more than 40 years, confirming that interfacial tension presents an intrinsic relationship with emulsion stability.^13,14^

However, because of the complexity of the phenomena that control the coalescence process, the effect of the interfacial properties on the emulsion stability is not yet fully understood. Also, most of the systems studied in the literature are produced with model fluids and do not reproduce the oilfield conditions.^1,10,15^ Furthermore, demulsifier efficiency and emulsion stability are commonly assessed by bottle tests, which can be considered laborious, time-consuming analysis, and do not consider the main factors controlling the kinetic stability of water–oil emulsions, such as interfacial tension.^14^

This study aims to assess how a commercial demulsifier affects the interfacial tension as a function of temperature and its concentration, enabling the application of this interfacial property as a sensitive parameter to evaluate the demulsifier’s effectiveness instead of the classical bottle tests. To establish this correlation, this study focused on the interfacial tension measurements using a spinning drop tensiometer and phase separation kinetics by a bottle test. This study can contribute to the microscopic understanding of the stability of emulsions, based on HLD concepts, however, applied for real production fluids composed of dead heavy oil (∼35,000 mPa·s, at 25 °C), produced water, and the commercial demulsifier used in a production system.

## 2 Materials and Methods

### 2.1 Materials

The tests were performed with two heavy crude oils, named “oil A” and “oil B”, and their respective produced water, “PW-A” and “PW-B”. As the focus is on the evaluation of the demulsifier on the interfacial rheological properties and emulsion stability, both systems were studied with and without the demulsifier. We highlight that the demulsifier studied is commercial, and its active component is an ethoxylated surfactant with 8–10 EO’s, currently used in oilfield production from where the crude oils and produced water were sampled. Other chemicals were also used (such as *n*-hexane and diesel) for pretreatment of the oil samples, as well as *n*-heptane, Attapulgite clay, and silica gel for SARA analysis.

### 2.2 Methods

#### 2.2.1 Pretreatment of Oil Samples

For emulsion stability discussion, it is necessary to obtain the physicochemical parameters of the crudes. The first step was dehydration of the water present in the crude oil samples. The procedure for dehydrating was performed by diluting the crude oil samples with 20 wt % of *n*-hexane to reduce the oil viscosities. After dilution, the oils were transferred to polypropylene centrifuge tubes and centrifuged at 60 °C, at 3756*g*, for 1 h. The water phase decanted to the bottom of the tube was removed with a syringe and a long needle. The *n*-hexane was removed by rotary evaporation at 70 °C. This procedure was reproduced on a large scale to obtain the necessary amount of dehydrated oil for the tests.

After the dehydration procedure, the water contents of oils A and B were measured by Karl Fisher titration, reaching average values of 0.15 ± 0.03 and 0.08 ± 0.02 wt %, respectively, being considered satisfactory for the beginning of the physicochemical characterization.

Because the crude oils are heavy, after the dehydration step, it was necessary to dilute them with 20 and 25 wt % of diesel for oils A and B, respectively. The dilution was necessary to make the viscosity of the dead oils approach the viscosity found in the oilfield, i.e., about 140 mPa·s, at 72 °C.

Although the pretreatment step involved the addition of a new component, changing a little bit the crude oil composition, it was the closest as possible to the real crude, at atmospheric pressure, i.e., without the light components. Additionally, it is important to highlight that the dehydration process was only performed to obtain a dehydrated crude sample for physicochemical characterization but not for the bottle tests.

#### 2.2.2 Compositional Analysis (SARA)

The compositional analysis of crude oil was carried out by separating the crude oil into four classes of components: Saturates, Aromatics, Resins, and Asphaltenes (SARA). The first step was precipitating asphaltenes by adding an excess of *n*-heptane in the oil sample (300 mL of *n*-heptane in 10 g of crude oil) under constant stirring for 24 h. The mixture was then filtered through cellulose acetate membranes, and the solid was dried in a laboratory oven at 120 °C until constant weight.

Because of technical limitations, the step of resin separation was adapted from ASTM 2007-11. In this step, approximately 100 g of Attapulgite clay (Brasilminas) was added to the maltenes fraction, and the mixture was stirred for two h at 25 °C using a magnetic stirrer. Then, the mixture was filtered under vacuum through filter paper (Whatman 42, Sigma-Aldrich). The clay mass containing the adsorbed resin fraction was dried in a laboratory oven at 150 °C until a constant weight. The resin mass was calculated by subtracting the clay mass.

The aromatic composition was separated from the residue of the resin extraction step, containing *n*-heptane, aromatics, and saturates. The separation was performed by elution of the mixture on a glass column packed with silica gel (35–70 mesh, Sigma-Aldrich) previously dried at 120 °C. After elution, the silica gel was poured into a beaker and dried at 150 °C until it reached a constant weight. The mass of aromatics was calculated by subtracting the initial mass of the dehydrated silica. The composition of the saturates was calculated by subtracting the mass of the previous fractions from the initial mass of the crude oil.

#### 2.2.3 Dynamic Viscosity

The dynamic viscosity of the dehydrated oils was measured in a rotational rheometer (HAAKE, MARS), using plate–plate geometry with a diameter of 35 mm and a gap of 1 mm.

Approximately 1 mL of sample was placed between the plates. The viscosities of oils A and B were measured in steps of 10 °C in the range of 55–115 °C, in the range of shear rate of 0–300 (1/s), for 5 min. Measurements were performed in duplicate.

#### 2.2.4 Density

Density measurements of oils A and B diluted in diesel and their respective produced waters were made by using a vibrating tube densimeter (DMA 4500M, Anton Paar). Densities were measured in duplicate, at temperatures of 30, 40, 50, and 60 °C, avoiding higher temperatures to prevent the formation of bubbles inside the equipment. For density values at temperatures above 70 °C, extrapolations of the fitted curves were made in the range of 30–60 °C. These results were used as input data for interfacial tension tests.

#### 2.2.5 Interfacial Tension

The interfacial tension measurement was carried out on a spinning drop tensiometer (SVT-20N, Dataphysics). The water was the filling fluid of the capillary tube, and an oil droplet was manually injected into the water phase by using a syringe/needle system. This tube was attached to the tensiometer rotor, and the spinning was started. The rotational speed value was defined based on the difference in density and the initial interfacial tension between the fluids.^16^

The interfacial tension measurement was calculated using the Laplace–Young (LY) equation (eq 3)­

3 $$\sigma =\frac{\Delta \rho \omega^{2}a^{3}}{2\alpha }$$

where σ is interfacial tension [N/m], Δρ is density difference [kg·m^–3^], ω is angular frequency [rad/s], *a* is the radius of the cap [m], and α is a shape parameter. Rotation speed is a parameter that depends on the difference in density between fluids and the initial interfacial tension of the system.^17^ The densities were measured and are reported in Table 3.

Interfacial tension was measured at some concentrations of demulsifier (0, 87, 300, 513, and 600 ppm) and temperatures (60, 64, 75, 86, and 90 °C). The analysis conditions were defined following the temperature range and demulsifier concentration values from the oilfield.

#### 2.2.6 Methodology to Evaluate the Emulsion Stability

The classic bottle test methodology was not applicable due to the high viscosity of the oil samples (∼35,000 mPa·s, @25 °C). Thus, the methodology was adapted to speed up the phase separation using a centrifuge.

For the tests, the crude oil and its produced water were initially placed into a beaker and heated in a water bath until reaching the desired temperature, and then the emulsion was prepared using a mechanical disperser (Turrax, T18, IKA), at 18,000 rpm, for 2.5 min. The water-cut was fixed at 50 wt % for all emulsion systems. After that, the demulsifier was transferred to the beaker and dispersed with Turrax for 2.5 min. The total volume of emulsion prepared for each system was approximately 35 mL.

After preparation, the emulsion was quickly transferred to 15 mL polypropylene centrifuge tubes and centrifuged under 3756*g*, at the same temperature at which the emulsion was prepared, for 1 h. The separated water was removed with a long needle attached to a syringe and weighed on an analytical balance. The bottle tests were performed on the same values of the concentration of the demulsifier and the temperature of the interfacial tension tests. The results were presented as mass percents of water separated as a function of the demulsifier concentration and temperature. The separation factor, *R*_sep_, is given by eq 4:

4 $$R_{\mathrm{sep}}=\frac{\mathrm{mass of separated water}}{\mathrm{mass of total water in the emulsion}}\times 100$$

#### 2.2.7 Experimental Design for Two Independent Variables

Experimental design is a method for modeling and determining the variables that affect the process’s outputs of interest. Although the goal of experimental design is to optimize the influential variables to achieve the best response of the process, the goal was not optimization necessarily, but the use of the Central Composite Rotatable Design (CCRD) methodology as a tool to assess how the temperature and demulsifier concentration affect the interfacial tension and the emulsion stability, also aiming to reduce the number of tests when compared to the experimental matrix, reduce analysis cost, time and amount of chemicals.^18,19^

The analysis of variance (ANOVA) was used to obtain a mathematical model with a significance level equal to 0.10 (*p* < 0.1). Terms with a *P*-value >0.1 are not significant and have little effect on the final equation and responses and are excluded from the final equation. The results of the mathematical model were evaluated in terms of coefficient of determination (*R* ^2^), Fisher variation ratio (*F*_value_), and *F*_table_.^19^

The analysis conditions were determined using a CCRD because it is an appropriate statistical tool to determine the effects of two independent factors (temperature and concentration) on the outputs of interest (interfacial tension and separation factor) according to a 2^2^ design.^20^ In this case, the experiment design consists of four tests in factorial conditions, four tests in axial conditions, and three center point repetitions, totaling 11 tests. The statistical analysis was performed using Protimiza Experimental Design software.^21^

## 3 Results and Discussion

Initially, we present the characterization of the fluids that compose both systems, which supports a phenomenological discussion. We then present and define the experimental matrix, which includes the independent variables and their respective levels (values), followed by responses (dependent variables) and discussion. Our objective is to provide a statistical interpretation of our results with the CCRD methodology.

### 3.1 Sample Characterization

The sample characterization of the crude samples was quantified by SARA, viscosity, and density analysis, according to Tables 1, 2, and 3, respectively.

**Table 1:** Results of the SARA Characterization of Crude Oils

	SARA composition (wt %)	
Compounds	Oil A	Oil B
Asphaltenes	22	19
Resins	33	27
Aromatics	12	7
Saturated	33	46

**Table 2:** Viscosity of Crude Oils A and B in the Range of 55–115 °C

	Viscosity (Pa·s)	
Temperature (°C)	Oil A	Oil B
55	9.1 ± 0.1	5.0 ± 0.2
65	3.6 ± 0.1	2.5 ± 0.1
75	1.8 ± 0.1	1.3 ± 0.1
85	1.0 ± 0.1	0.7 ± 0.1
95	0.5 ± 0.1	0.4 ± 0.1
105	0.3 ± 0.1	0.3 ± 0.1
115	0.2 ± 0.1	0.2 ± 0.1

**Table 3:** Density of Crude Oil and Produced Water A and B

	Density (g/cm^3^) (±1.10^–4^)			
Temperature (°C)	Oil A	Oil B	PW-A	PW-B
60	0.9278	0.9286	1.0413	1.0489
64	0.9251	0.9260	1.0394	1.0468
75	0.9180	0.9188	1.0342	1.0412
86	0.9108	0.9116	1.0289	1.0355
90	0.9081	0.9090	1.0270	1.0334

Although the oils come from the same reservoir, crude oil B presents fewer amounts of resins, asphaltenes, and aromatics than crude oil A, but a greater amount of saturates. Several authors have concluded that asphaltenes and resins are the compounds that most affect the stability of crude oil emulsions and, consequently, the kinetics of phase separation.^22–24^ Once formed, nanoaggregates of asphaltenes can adsorb at the water–oil interface, forming a viscoelastic film around the dispersed droplets, which provides great stability for emulsions.^10,25^

According to Table 2, oil A is more viscous than oil B below 95 °C. However, since viscosity is related to the continuous phase, a greater water recovery is expected for the system composed of oil B because the lower the viscosity, the more frequent the effective droplet–droplet collisions, increasing the coalescence rate and, consequently, the phase separation. Reduction in the viscosity of the continuous phase facilitates the diffusion of water droplets to the bottom of the polypropylene centrifuge tubes during centrifugation.

The high viscosities of crude oils are related to their chemical compositions, mainly the concentration of heavier petroleum fractions, such as asphaltenes and resins^26^ The SARA results (Table 1) indicate that oil A exhibits a higher concentration of these components, justifying its higher viscosity under the same conditions.

### 3.2 CCRD Methodology

Table 4 shows the independent variables concentration (*x*_1_), temperature (*x*_2_), and levels according to the CCRD 2^2^ planning with three repetitions at the central point (300 ppm; 75 °C).

**Table 4:** Independent Variables and Levels Were Defined for the CCRD Analysis

Concentration (ppm) (x_1_)	Temperature (°C) (*x*_2_)
87	64
513	64
87	86
513	86
0	75
600	75
300	60
300	90
300	75
300	75
300	75

The responses *Y*_1A_, *Y*_2A_, *Y*_1B_, and *Y*_2B_ represent the separation factor and interfacial tension for systems A and B, respectively, and the results are shown in Table 5.

**Table 5:** Central Composite Rotatable Design (CCRD) 2^2^ Planning

System A (Oil A + PW-A)			System B (Oil B + PW-B)
(*Y*_1A_) Separation factor (%)	(*Y*_2A_) Interfacial tension (mN/m)	(*Y*_1B_) Separation factor (%)	(*Y*_2B_) Interfacial tension (mN/m)
0.0	1.0	0.0	1.8
7.9	1.0	22.4	1.0
3.3	3.1	1.8	3.0
14.0	1.8	54.8	2.1
0.0	12.9	0.0	13.0
16.1	1.2	48.1	1.3
1.7	1.0	1.2	1.0
9.6	1.9	28.4	2.3
17.0	1.3	41.0	1.1
20.3	1.1	44.8	1.2
20.6	1.4	39.7	1.4

The response surface models *Y*_1A_, *Y*_2A_, *Y*_1B_, and *Y*_2B_; *F*_value_; *F*_table_; and correlation coefficient (*R* ^2^) obtained from ANOVA are shown in Table 6, and Figure 1 illustrates the predicted versus experimental values for each response surface model. *F*_value_ and *F*_table_ are statistical terms from ANOVA analysis, used to compare the variations between the two samples.^20,21^ All of the CCRD analyses were performed using the Protimiza Experimental Design Software.

**Figure 1:**
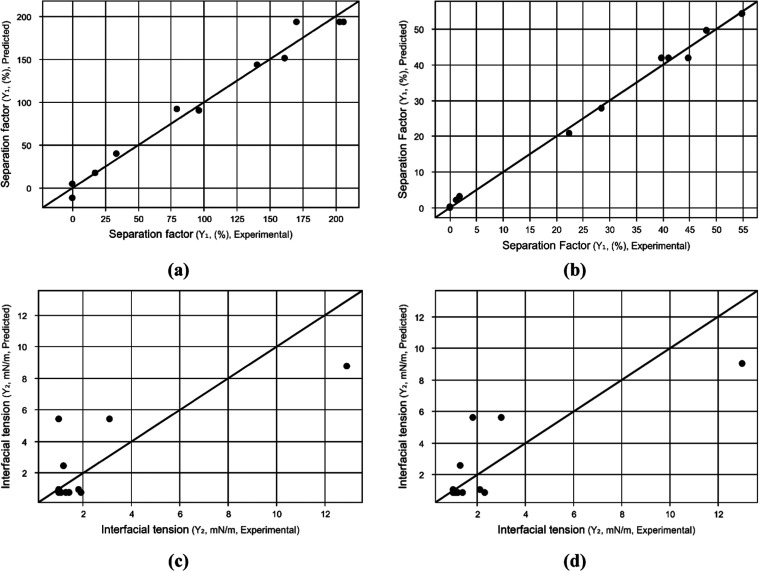
Predicted (black line) versus experimental values (black points) for (a) *Y*_1A_, (b) *Y*_1B_, (c) *Y*_2A_, and (d) *Y*_2B_.

**Table 6:** Analysis of Variance for *Y*_1A_, *Y*_2A_, *Y*_1B_, and *Y*_2B_

Response surface model	Correlation coefficient *R* ^2^ (%)	*F*_value_	*F*_table_
*Y*_1A_ = 19.30 + 5.17 *x*_1_ – 5.76 *x*_1_^2^ + 2.57 *x*_2_ – 6.96 *x*_2_^2^	98.0	71.6	3.9
*Y*_2A_ = 0.76 – 2.23 *x*_1_ + 2.42 *x*_1_^2^	62.1	6.5	4.3
*Y*_1B_ = 41.83 + 17.93 *x*_1_ – 8.81 *x*_1_^2^ + 9.08 *x*_2_ – 13.44 *x*_2_^2^ + 7.65 *x*_1_ *x*_2_	99.5	198.4	3.5
Y_2B_ = 0.86–2.28 *x*_1_ + 2.47 *x*_1_^2^	65.2	7.5	3.1

According to the ANOVA evaluation, the response surface model is statistically significant since the correlation coefficient (*R* ^2^) is good enough and *F*_value_ is at least 3–5 times greater than *F*_table_.^21^

Based on Table 6 and Figure 1, the high values of *R* ^2^ equal to 98.0 and 99.5 for surface models *Y*_1A_ and *Y*_1B_, respectively, suggest that the models are statistically significant and the experimental values are close to the predicted ones because both temperature (*x*_2_^2^) and concentration (*x*_1_^2^) proved highly effective in predicting the separation factor. Additionally, the interaction between temperature and concentration (*x*_1_ *x*_2_) impacts *Y*_1B_, making the mathematical model more robust. On the other hand, for interfacial tension responses (*Y*_2A_ and *Y*_2B_), only the concentration (*x*_1_^2^) was significant, making the mathematical model less robust. A hypothesis of the impact of the interaction term (*x*_1_ *x*_2_) in *Y*_1B_, but not in *Y*_1A_, could be associated with SARA composition, but additional studies are needed.

Temperature acts not only on the characteristics of the interfacial film established at the oil/water interface but also reduces the viscosity of the continuous phase and, consequently, increases the frequency of effective drop–drop collisions.^26^

Commercial demulsifiers can have different formulations, including organic solvents such as alcohol, toluene, xylene, naphtha, polymers, and ionic or nonionic surfactants. The efficiency of demulsifiers based on ethoxylated surfactants, as in our study, can be significantly influenced by temperature; that is, as the system temperature increases, the EO surfactant becomes less soluble in water and more soluble in the oil phase, as a consequence of the breaking of hydrogen bonds between POE groups and water. Their molecular structure can be adapted to cover different types of oil, because they interact differently with the components of the crude, influencing their demulsification performance.^27^

According to Marquez et al.,^10,12^ the presence of a demulsifier reduces the interfacial tension gradients because the diffusive surfactant exchanges with the interface are very fast, and the Gibbs–Marangoni effect that counters the destabilization of emulsion is almost negligible.

Comparing response surfaces between the *R*_sep_ and interfacial tension, our results indicate an inverse correlation at the same range of demulsifier concentration, as observed by Marquez et al.^1,12^ The emulsions composed of oil A achieved a maximum *R*_sep_ in the range of 250–550 ppm of demulsifier at 70–85 °C, whereas a minimum interfacial tension was found between 300 and 500 ppm (Figure 2). For the system composed of oil B, we observed a maximum *R*_sep_ in the range of 270–650 ppm at 70–95 °C, whereas a minimum interfacial tension was found between 300 and 500 ppm (Figure 3).^28,29^

**Figure 2:**
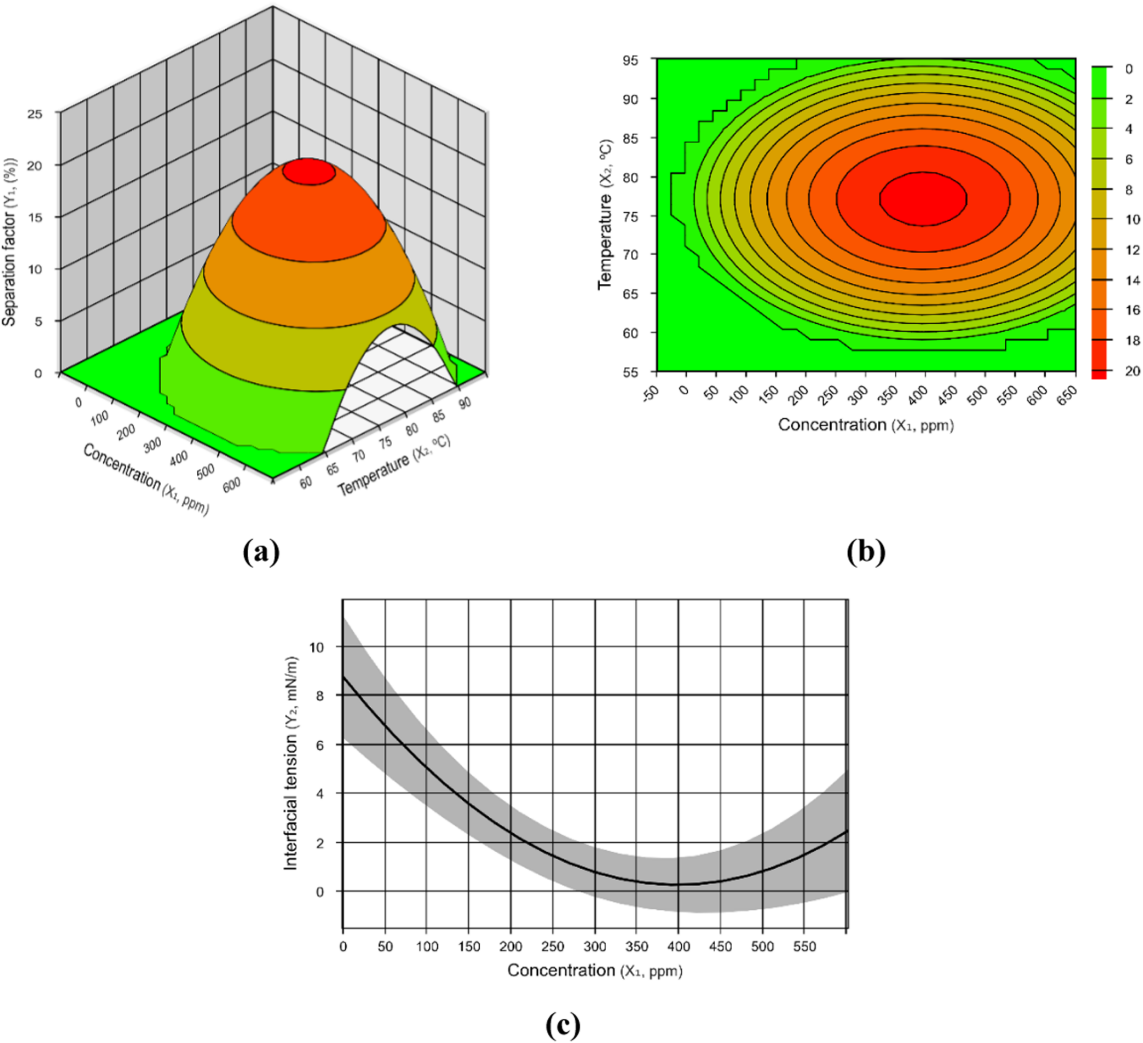
Results for emulsions with oil A. (a) Separation factor (*Y*_1A_) response surface as a function of temperature and demulsifier concentration; (b) level sets of the surface graph presented in (a); (c) interfacial tension (*Y*_2A_) response as a function of demulsifier concentration. Orange and red colors represent the optimum range of conditions for water–oil separation.

**Figure 3:**
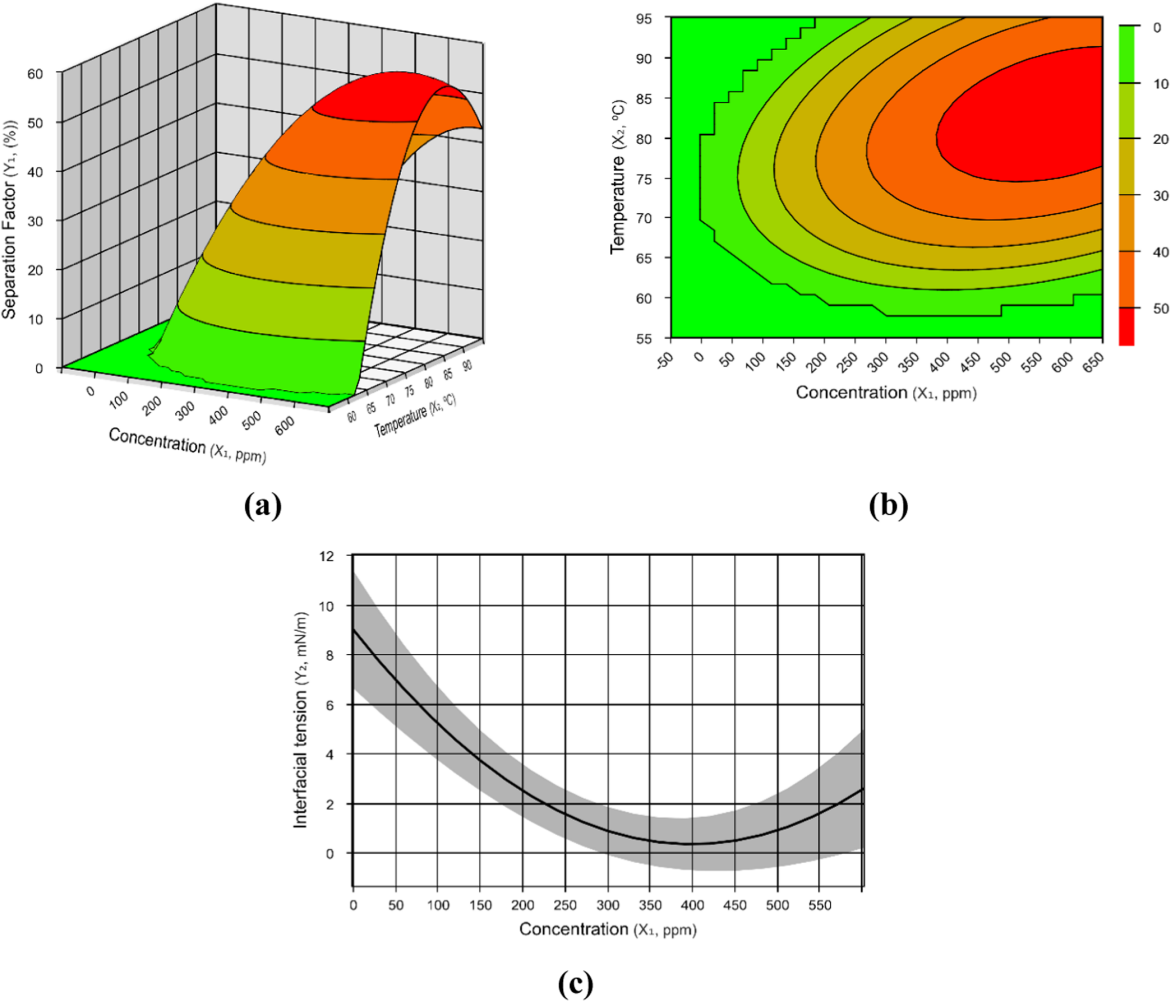
Results for emulsions with oil B. (a) Separation factor (*Y*_1B_) response surface as a function of temperature and demulsifier concentration; (b) level sets of the surface graph presented in panel (a); (c) interfacial tension (*Y*_2B_) response as a function of demulsifier concentration. Orange and red colors represent the optimum range of conditions for the water–oil separation.

At optimum formulation (HLD = 0), the interfacial tension exhibits a deep minimum and efforts have been put toward explaining how these findings could contribute to decreasing the stability of emulsions.^3,11,30^ The inverse correlation between *R*_sep_ and interfacial tension can occur as a result of disturbances to the system caused by stretching on the surface of the drops and generating interfacial tension gradients that facilitate the migration/adsorption of surfactant molecules. Thus, as new regions of high interfacial tension are created at the interface by the stretching process, they are quickly occupied by more demulsifier molecules, which dissipate the tension gradient and enhance film drainage (thinning of the film between two drops).^5,7^

Our results indicated that the effect of increasing demulsifier concentration was more significant for the increase in *R*_sep_ compared to the temperature. The rise in demulsifier concentration dropped the interfacial tension gradients by promoting fast diffusive exchanges at the interface. It is known that the molecules of demulsifiers are larger than natural surfactants, which makes them more able to replace the natural surfactants at the interface. This indicates a slack structure of the interfacial film that coalesces rapidly.^30,31^ Therefore, the deep minimum of interfacial tension associated with more drop–drop collisions favors the coalescence rate and contributes to the phase separation and decrease in the *R*_sep_.

The increase in temperature improved the *R*_sep_ by reducing the crude oil’s viscosity, favoring the drop–drop collisions. It is known that lower temperatures lead to stronger attractive forces between the two phases of emulsion by increasing the cohesion of intermolecular species and increasing interfacial tension.^26^ Because the efficiency of ethoxylated surfactants can be significantly influenced by temperature, it is assumed that the demulsifier becomes more soluble in oil than water, i.e., changing the surfactant partition coefficient, consequently reducing its efficiency in the demulsification process.

Regarding SARA characterization, asphaltene concentration plays an important role in stabilizing emulsions, i.e., the thickness of the interfacial film increases as the asphaltene concentration in the system increases, providing a better stability for emulsions^25,32^ Our results showed agreement with the literature since oil B has less asphaltene than oil A, the system composed of oil B recovered more water, for the same temperature conditions and demulsifier concentration.

Interfacial tension presented a significant role in the evaluation of the selected demulsifier performance for crude oil dehydration and improved phase separation, aside from being an independent parameter of the physical-chemical characterization of the oil/water system.

## 4 Conclusions

The CCRD methodology was useful in determining the effect of the temperature and demulsifier concentration on interfacial tension and emulsion stability at optimal separation conditions. Because the concentration range for a maximum separation coincides with the minimum interfacial tension at 300–500 ppm for both systems studied, the results suggest that interfacial tension may be used as a parameter to evaluate emulsion stability and demulsifier efficiency. There was an intrinsic relationship between the interfacial tension and phase separation, which helps us conclude that this interfacial property can be adequately related to the HLD and demulsifier efficiency.

We also highlight that the study was performed for real production fluids, i.e., composed of real crude oils, produced water, and a commercial demulsifier already used in the oilfields. Our findings corroborated the fact that the previous interfacial tension understandings were the same as those reached for simple systems at optimum formulation. Also, trends in correlations and the mathematical model may apply to other w/o systems, but the optimum conditions are shown to be dependent on the oil’s composition, produced water, and consequently, the surfactant formulation.

The impact of well pressure and temperature on the emulsion stability and demulsifier efficiency, simulating the reservoir conditions inside a PVT cell, will be studied to improve our outcomes.
